# Association between white blood cell count to hemoglobin ratio and risk of in-hospital mortality in patients with lung cancer

**DOI:** 10.1186/s12890-023-02600-7

**Published:** 2023-08-18

**Authors:** Tingting Gao, Yurong Wang

**Affiliations:** 1 Department of Comprehensive Medical, Shanxi Bethune Hospital, Shanxi Academy of Medical Sciences, Tongji Shanxi Hospital, Third Hospital of Shanxi Medical University, Taiyuan, 030032 P.R. China; 2 Department of Clinical Laboratory, Nanjing Jiangbei Hospital Affiliated to Nantong University, 552 Geguan Road, Jiangbei New District, Nanjing, Jiangsu 210048 P.R. China

**Keywords:** Association, White blood cell count to hemoglobin ratio, In-hospital mortality, Intensive care unit, Lung cancer

## Abstract

**Background:**

The objective of this study was to investigate the association between white blood cell count to hemoglobin ratio (WHR) and risk of in-hospital mortality in patients with lung cancer.

**Methods:**

In this retrospective cohort study, the medical records of patients with lung cancer were retrieved from the electronic ICU (eICU) Collaborative Research Database between 2014 and 2015. The primary outcome was in-hospital mortality. The secondary outcome was the length of stay in intensive care unit (ICU). The cut-off value for the WHR was calculated by the X-tile software. The Cox model was applied to assess the association between WHR and in-hospital mortality among patients with lung cancer and the linear regression model was used to investigate the association between WHR and length of ICU stay. Subgroup analyses of age (< 65 years or >  = 65 years), Acute Physiology and Chronic Health Evaluation (APACHE) score (< 59 or >  = 59), gender, ventilation (yes or no), and vasopressor (yes or no) in patients with lung cancer were conducted.

**Results:**

Of the 768 included patients with lung cancer, 153 patients (19.92%) died in the hospital. The median total follow-up time was 6.88 (4.17, 11.23) days. The optimal cut-off value for WHR was 1.4. ICU lung cancer patients with WHR >  = 1.4 had a significantly higher risk of in-hospital mortality [Hazard ratio: (HR): 1.65, 95% confidence interval (CI): 1.15 to 2.38, *P* = 0.007) and length of stay in ICU (HR: 0.63, 0.01, 95% CI: 1.24 to 0.045, *P* = 0.045). According to the subgroup analysis, WHR was found to be associated with in-hospital mortality in patients with higher APACHE score (HR: 1.60, 95% CI: 1.06 to 2.41, *P* = 0.024), in male patients (HR: 1.87, 95% CI: 1.15 to 3.04, *P* = 0.012), and in patients with the treatment of ventilation (HR: 2.33, 95% CI: 1.49 to 3.64, *P* < 0.001).

**Conclusion:**

This study suggests the association between WHR and risk of in-hospital mortality in patients with lung cancer and length of stay, which indicates the importance of attention to WHR for patients with lung cancer.

**Supplementary Information:**

The online version contains supplementary material available at 10.1186/s12890-023-02600-7.

## Background

Lung cancer is one of the most commonly diagnosed cancers and the leading cause of mortality worldwide, accounting for approximately 18% of all cancer mortality [1]. Comprehensive screening and advances in therapeutic strategies have improved the survival of lung cancer patients [2]. However, Lung cancer patients usually require admission to an intensive care unit (ICU) for invasive monitoring or treatment due to the nature of the disease and aggressive treatments [3]. Although progressive improvements have been made to improve the prognosis of lung cancer patients admitted to ICUs, the mortality rate remains extremely high. The in-hospital mortality rate for lung cancer patients is estimated to be 60% [4]. Therefore, it is important for clinicians to recognize the factors associated with a high risk of mortality in lung cancer patients.

Previous evidence suggests that chronic low-level inflammation is an important factor affecting cancer development and prognosis [5]. The markers of the systemic inflammatory response, such as platelet to lymphocyte ratio (PLR) and neutrophil to lymphocyte ratio (NLR) have been shown to play an important role in the progression and prognosis of patients with lung cancer [6, 7]. However, most of the studies have focused on specific subgroups of white blood cells (WBC) [8]. WBCs, as a complete cell type in human blood, have been reported as one of the most important components of the immune system [9]. WBC level has been reported to be associated with early mortality in epithelial ovarian cancer [10]. Anemia, a condition of insufficient oxygen-carrying capacity, defined as a low level of hemoglobin (HGB) in the blood, is a common problem in the ICU [11]. Low levels of HGB have been reported as the cause of poor oxygen delivery to the tumor [12]. A previous study demonstrated that low HGB levels lead to an increased risk of lung cancer mortality [13]. Recently, WBC to HGB ratio (WHR) has been developed to characterize immune inflammatory states and anoxic microenvironments and has been found to be a prognostic factor for malignant tumors such as hepatocellular carcinoma, gastric adenocarcinoma, and bladder cancer [8, 14, 15]. However, to the best of our knowledge, no study has examined the association between WHR and in-hospital mortality in patients with lung cancer in the ICU. Evaluation of simple and available serum indexes may provide guidance for clinical workers in the management of lung cancer patients in the ICU.

Herein, the purpose of this study was to investigate the association between WHR and the risk of in-hospital mortality in patients with lung cancer.

## Methods

### Study design and patients

In this retrospective cohort study, data were from the electronic ICU (eICU) Collaborative Research Database: https://eicu-crd.mit.edu/. The Collaborative Research Database is a multi-center critical care database containing data from more than 200 000 ICU admissions from 208 hospitals across the United States between 2014 and 2015 [16]. Included criteria were: (1) age ≥ 18 years; (2) diagnosed with lung cancer; and (3) admitted to the ICU for more than 24 h. Excluded criteria were: (1) lack of key data such as WBC, and HGB; (2) loss of survival data. Due to the retrospective nature of the study, it was not necessary to obtain informed consent. As our data were obtained from a public database, the approval of our hospital’s ethics committee was not required.

### Data extraction

The extracted information of the patients included: (1) baseline characteristics: age (years), race, interventions, tumor types, body mass index (BMI, kg/m^2^), heart rate, blood pressure, respiratory rate, systolic blood pressure (SBP, mmHg), diastolic blood pressure (DBP, mmHg), and temperature (°C); (2) comorbidities: coronary artery disease (CAD), congestive heart failure (CHF), atrial fibrillation (AF), renal failure (RF), diabetes, hypertension, and chronic kidney disease; (3) scoring systems: Acute Physiology and Chronic Health Evaluation (APACHE) score; (4) laboratory parameters: creatinine (mg/dL), blood urea nitrogen (BUN, mg/dL), glucose (mg/dl), bicarbonate (mmol/L), sodium (mmol/L), potassium (mmol/L), chloride (mmol/L), HGB (g/dl); (5) inflammatory biomarker: WHR and PLR. Data extraction was performed during the first 24 h of ICU admission.

Races were grouped into White and other. Interventions were recorded as mechanical ventilation, vasopressors, rapid resolution therapy (RRT), sedatives, and opioids. Tumor types were identified as primary lung cancer, adenocarcinoma, squamous cell carcinoma, and unknown. APACHE II consists of the acute physiological score, age score, and chronic health score, with a score ranging from 0 to 71. The higher the score, the more severe the disease. The comorbidities were collected for analysis based on the recorded ICD codes in the eICU Collaborative Research Database.

### Definitions and outcomes

The study included adult patients with a diagnosis of lung cancer according to the ninth or tenth revision of the International Classification of Diseases (ICD-9/10) at the time of admission. The WHR was calculated by WBC/HGB. PLR was platelet count/lymphocytes count.

The primary outcome was in-hospital mortality among patients with lung cancer. The secondary outcome was the length of stay in ICU. In-hospital mortality was defined as death occurring before hospital discharge. Length of stay in the ICU was defined as the number of days spent in the ICU. Follow-up was conducted by consulting hospitalization records. The median total follow-up time was 6.88 (4.17, 11.23) days.

### Statistical analysis

Continuous data with normal distribution were expressed as means +—standard deviation (SD), and comparison between groups was used T-test. Continuous data in skew distribution were expressed as median and quartile [M (Q_1_, Q_3_)] and compared using the independent-sample Wilcoxon rank sum test. Categorical data were presented as n (%) and analyzed using the chi-square test. Missing values are interpolated using random forest interpolation. The missing values before and after interpolation were compared between groups as sensitivity analysis. Sensitivity analysis before and after interpolation is shown in Supplementary Table 1.

The optimal cut-off value for WHR and PLR was 1.4 and 61.4, respectively. The univariate Cox model for assessing the association between WHR and in-hospital mortality among ICU patients with lung cancer and the univariate linear regression model for assessing the association between WHR and length of ICU stay were (model 1) performed to select covariates for adjustment, and covariates with a *P* value of less than 0.05 were considered potential confounders. In the multivariable Cox model analysis, model 2 adjusted for age, gender, BMI, and race, and model 3 adjusted for age, gender, race, ICU stay time, BMI, heart rate, SBP, BUN, potassium, chloride, RF, ventilation, vasopressor, APACHE score, and PLR; In the multivariable linear regression model analysis, model 2 adjusted for age, sex, race, BMI, and model 3 adjusted for APACHE score, BMI, heart rate, glucose, CAD, CHF, AF, RF, hypertension, RRT, ventilation, and vasopressor. To determine whether the same indicator was applicable across the subgroups, we carried out a subgroup analysis of age (< 65 years or >  = 65 years), APACHE score (< 59 or >  = 59), gender, ventilation (yes or no), and vasopressor (yes or no) in ICU patients with lung cancer.

The hazard ratio (HR) with 95% confidence intervals (95% CI) was reported, and statistical significance was assessed at the 0.05 level. The optimal cut-off values for WHR and PLR were selected using the X-tile software. R version 4.2.0 (2022–04-22 ucrt) was used for statistical analysis.

## Results

### Characteristics of included patients

**Fig. 1:**
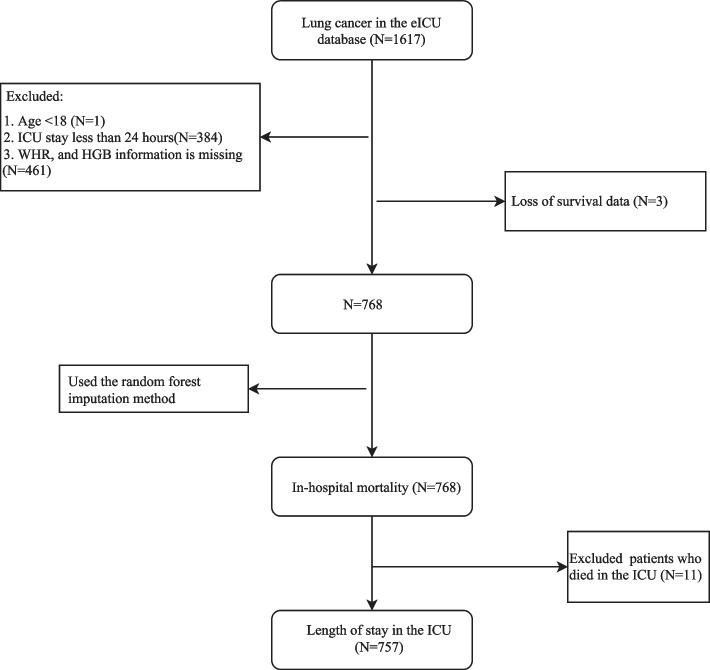
A flow chart of patents selection

A total of 768 patients with lung cancer were selected for this study. A flow chart showing how participants were selected is shown in Fig. 1. In-hospital mortality occurred in 153 patients (19.92%). The mean age is 68.17 ± 10.39 years. The majority of patients [570 (74.22%)] presented with primary lung cancer. The median ICU stay was 4111.00 (2517.50, 7089.00) minutes. There were significant differences between patients with in-hospital mortality and patients without in-hospital mortality in heart rate, SBP, DBP, creatinine, BUN, potassium, RF, ventilation, vasopressor, APACHE score, WHR, PLR, and ICU stay time (each *P* < 0.05). The characteristics of the included patients are described in Table 1.

**Table 1:** Characteristics of included patients

		In-hospital mortality			
Variables	Total (*n* = 768)	No (*n* = 615)	Yes (*n* = 153)	Statistics	*P*
WHR, n (%)				χ^2^ = 40.478	< 0.001
< 1.4	571 (74.35)	488 (79.35)	83 (54.25)		
> = 1.4	197 (25.65)	127 (20.65)	70 (45.75)		
PLR, n (%)				χ^2^ = 21.601	< 0.001
< 1.4	603 (78.52)	504 (81.95)	99 (64.71)		
> = 1.4	165 (21.48)	111 (18.05)	54 (35.29)		
Age, year, Mean ± SD	68.17 ± 10.39	68.03 ± 10.31	68.75 ± 10.73	t = -0.76	0.446
Race, n (%)				χ^2^ = 0.051	0.822
White	648 (84.38)	518 (84.23)	130 (84.97)		
Other	120 (15.63)	97 (15.77)	23 (15.03)		
BMI, kg/m^2^, Mean ± SD	26.01 ± 6.43	26.03 ± 6.34	25.92 ± 6.79	t = 0.19	0.852
Heart rate, times/minute, Mean ± SD	99.60 ± 23.46	97.69 ± 23.04	107.25 ± 23.62	t = -4.57	< 0.001
Respiratory rate, breaths/minute, Mean ± SD	21.56 ± 6.43	21.34 ± 6.32	22.43 ± 6.81	t = -1.88	0.061
SBP, mmHg, Mean ± SD	121.76 ± 28.10	123.01 ± 28.47	116.73 ± 26.09	t = 2.48	0.013
DBP, mmHg, Mean ± SD	68.89 ± 17.18	69.56 ± 16.86	66.21 ± 18.23	t = 2.17	0.031
Temperature, °C, Mean ± SD	36.79 ± 0.74	36.79 ± 0.69	36.76 ± 0.92	t = 0.32	0.747
Creatinine, mg/dL, M (Q_1_, Q_3_)	0.90 (0.69, 1.30)	0.89 (0.69, 1.23)	1.03 (0.70, 1.52)	Z = 2.482	0.013
BUN, mg/dL, M (Q_1_, Q_3_)	19.00 (13.00, 29.00)	18.00 (13.00, 26.00)	25.00 (17.00, 38.00)	Z = 5.553	< 0.001
Glucose, mg/dl, M (Q_1_, Q_3_)	133.00 (106.00, 168.00)	132.00 (106.00, 161.00)	138.00 (107.00, 180.00)	Z = 1.344	0.179
Bicarbonate, mmol/L, Mean ± SD	25.41 ± 5.27	25.47 ± 5.08	25.17 ± 5.99	t = 0.56	0.574
Sodium, mmol/L, Mean ± SD	136.16 ± 5.89	136.24 ± 5.85	135.85 ± 6.05	t = 0.73	0.467
Potassium, mmol/L, Mean ± SD	4.19 ± 0.71	4.16 ± 0.68	4.33 ± 0.79	t = -2.46	0.015
Chloride, mmol/L, Mean ± SD	100.91 ± 6.88	101.12 ± 6.99	100.05 ± 6.35	t = 1.72	0.086
CAD, n (%)				χ^2^ = 2.163	0.141
No	491 (63.93)	401 (65.20)	90 (58.82)		
Yes	277 (36.07)	214 (34.80)	63 (41.18)		
CHF, n (%)				χ^2^ = 0.000	0.985
No	683 (88.93)	547 (88.94)	136 (88.89)		
Yes	85 (11.07)	68 (11.06)	17 (11.11)		
AF, n (%)				χ^2^ = 3.810	0.051
No	638 (83.07)	519 (84.39)	119 (77.78)		
Yes	130 (16.93)	96 (15.61)	34 (22.22)		
RF, n (%)				χ^2^ = 15.271	< 0.001
No	666 (86.72)	548 (89.11)	118 (77.12)		
Yes	102 (13.28)	67 (10.89)	35 (22.88)		
Diabetes, n (%)				χ^2^ = 0.111	0.739
No	659 (85.81)	529 (86.02)	130 (84.97)		
Yes	109 (14.19)	86 (13.98)	23 (15.03)		
Hypertension, n (%)				χ^2^ = 0.743	0.389
No	608 (79.17)	483 (78.54)	125 (81.70)		
Yes	160 (20.83)	132 (21.46)	28 (18.30)		
Chronic kidney disease, n (%)				χ^2^ = 0.025	0.876
No	731 (95.18)	585 (95.12)	146 (95.42)		
Yes	37 (4.82)	30 (4.88)	7 (4.58)		
Ventilation, n (%)				χ^2^ = 36.636	< 0.001
No	585 (76.17)	497 (80.81)	88 (57.52)		
Yes	183 (23.83)	118 (19.19)	65 (42.48)		
RRT, n (%)				-	0.768
No	750 (97.66)	601 (97.72)	149 (97.39)		
Yes	18 (2.34)	14 (2.28)	4 (2.61)		
Vasopressor, n (%)				χ^2^ = 32.960	< 0.001
No	637 (82.94)	534 (86.83)	103 (67.32)		
Yes	131 (17.06)	81 (13.17)	50 (32.68)		
APACHE score, M (Q_1_,Q_3_)	58.00 (46.00, 75.00)	55.00 (44.00, 70.00)	74.00 (58.00, 88.00)	Z = 7.884	< 0.001
Sedative, n (%)				χ^2^ = 3.180	0.075
No	732 (95.31)	582 (94.63)	150 (98.04)		
Yes	36 (4.69)	33 (5.37)	3 (1.96)		
Opioid, n (%)				χ^2^ = 2.163	0.141
No	491 (63.93)	401 (65.20)	90 (58.82)		
Yes	277 (36.07)	214 (34.80)	63 (41.18)		
Tumor type, n (%)				χ^2^ = 3.605	0.307
Primary lung cancer	570 (74.22)	462 (75.12)	108 (70.59)		
Denocarcinoma	68 (8.85)	50 (8.13)	18 (11.76)		
Squamous cell carcinoma	61 (7.94)	51 (8.29)	10 (6.54)		
Others	69 (8.98)	52 (8.46)	17 (11.11)		
ICU stay time, min, M (Q_1_, Q_3_)	4111.00 (2517.50, 7089.00)	3867.00 (2434.00, 6835.00)	5309.00 (3182.00, 8956.00)	Z = 4.205	< 0.001

*WHR* white blood cells/hemoglobin, *PLR* platelet count/lymphocytes count, *BMI* body mass index, *SBP* systolic blood pressure, *DBP* diastolic blood pressure, *BUN* blood urea nitrogen, *CAD* coronary artery disease, *CHF* congestive heart failure, *AF* Atrial fibrillation, *RF* Renal failure, *RRT* rapid resolution therapy, *APACHE* Acute Physiology and Chronic Health Evaluation, *ICU* intensive care unit

t: t test; Z: rank sum test; χ^2^: chi-square test; -: Fisher exact probability; Mean ± SD: means +—standard deviation; M: Median; Q_1_: 1st Quartile; Q_3_: 3st Quartile

### Association between WHR, PLR and risk of in-hospital mortality in patients with lung cancer

The univariate analysis of the Cox model showed that the WHR was associated with the risk of in-hospital mortality in patients with lung cancer (HR: 2.08, 95% CI: 1.51 to 2.87, *P* < 0.001). Model 3 also indicated that ICU patients with lung cancer with WHR >  = 1.4 had a significantly higher risk of in-hospital mortality (HR: 1.65, 95% CI: 1.15 to 2.38, *P* = 0.007). However, an increase in PLR was not related to the risk of in-hospital mortality in patients with lung cancer (HR: 1.30, 95% CI: 0.88 to 1.93, *P* = 0.188). Associations between WHR, PLR and risk of in-hospital mortality in ICU patients with lung cancer are presented in Table 2.

**Table 2:** Association between WHR, PLR and risk of in-hospital mortality in ICU patients with lung cancer

	Model 1		Model 2		Model 3	
Variables	HR (95% CI)	*P*	HR (95% CI)	*P*	HR (95% CI)	*P*
WHR, n (%)						
< 1.4	Ref		Ref		Ref	
> = 1.4	2.08 (1.51, 2.87)	< 0.001	2.03 (1.47, 2.81)	<0 .001	1.65 (1.15, 2.38)	0.007
PLR, n (%)						
< 1.4	Ref		Ref		Ref	
> = 1.4	1.75 (1.25, 2.44)	0.001	1.74 (1.23, 2.47)	0.002	1.30 (0.88, 1.93)	0.188

Model 1 was an unadjusted model; model 2 adjusted for age, gender, race, and BMI; model 3 adjusted for age, gender, race, ICU stay time, BMI, heart rate, SBP, BUN, potassium, chloride, RF, ventilation, vasopressor, and APACHE score, WHR additionally adjusted for PLR, and PLR additionally adjusted for WHR

*WHR* white blood cells/hemoglobin, *PLR* platelet count/lymphocytes count, *HR* hazard ratio, *CI* confidence interval, *ICU* intensive care unit, Ref reference

### Association between WHR and length of stay in patients with lung cancer

The result demonstrated that high WHR was related to the length of stay in ICU in patients with lung cancer (HR: 0.63, 0.01, 95% CI: 1.24 to 0.045, *P* = 0.045) (Table 3).

**Table 3:** Association between WHR, PLR and length of stay in ICU in ICU patients with lung cancer

	Model 1		Model 2		Model 3	
Variables	β (95% CI)	*P*	β (95%CI)	*P*	β (95% CI)	*P*
WHR, n (%)						
< 1.4	Ref		Ref		Ref	
> = 1.4	1.26 (0.59, 1.92)	< 0.001	1.27 (0.6, 1.93)	<0 .001	0.63 (0.01, 1.24)	0.045
PLR, n (%)						
< 1.4	Ref		Ref		Ref	
> = 1.4	0.75 (0.03, 1.46)	0.040	0.72 (0.01, 1.43)	0.049	0.59 (-0.06, 1.24)	0.077

Model 1 was an unadjusted model; model 2 adjusted for age, gender, race, and BMI; model 3 adjusted for APACHE score, BMI, heart rate, glucose, CAD, CHF, AF, RF, hypertension, RRT, ventilation, and vasopressor

*WHR* white blood cells/hemoglobin, *PLR* platelet count/lymphocytes count, *CI* confidence interval, *ICU* intensive care unit; Ref: reference

### Subgroup analysis association between WHR and risk of in-hospital mortality in patients with lung cancer

**Fig. 2:**
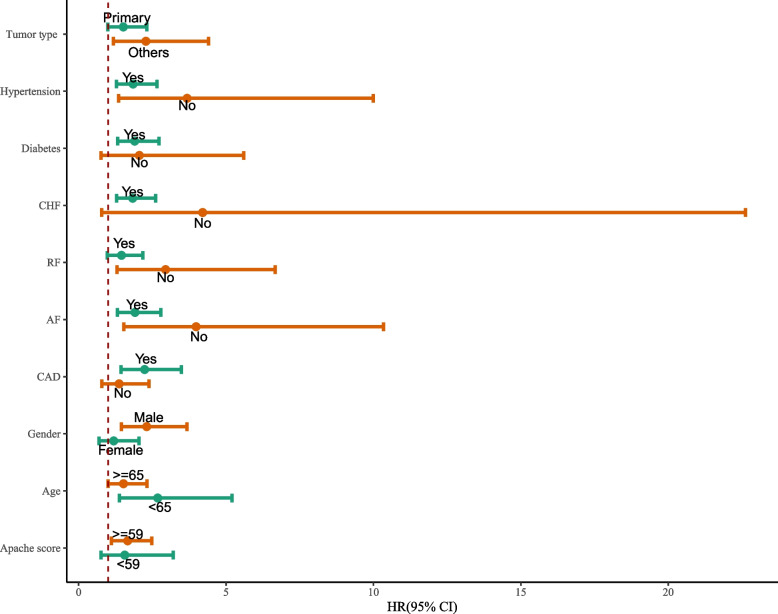
Subgroup analysis association between WHR and risk of in-hospital mortality in ICU patients with lung cancer

According to the subgroup analysis of age, WHR was found to be associated with the risk of in-hospital mortality in patients with lung cancer with age < 65 years (HR: 2.78, 95% CI: 1.44 to 5.39, *P* = 0.002) and age >  = 65 years (HR: 1.61, 95% CI: 1.04 to 2.49, *P* = 0.033). The high WHR was also related to a higher risk of in-hospital mortality in patients with higher APACHE score (HR: 1.60, 95% CI: 1.06 to 2.41, *P* = 0.024), in male patients (HR: 1.87, 95% CI: 1.15 to 3.04, *P* = 0.012), and in patients with the treatment of ventilation (HR: 2.33, 95% CI: 1.49 to 3.64, *P* < 0.001). WHR was also associated with the risk of in-hospital mortality in patients with (HR: 1.99, 95% CI: 1.29 to 3.06, *P* = 0.002) or without the treatment of vasopressor (HR: 2.25, 95% CI: 1.13 to 4.46, *P* = 0.021). Subgroup analysis association between WHR and risk of in-hospital mortality in patients with lung cancer is shown in Fig. 2.

## Discussion

As one of the leading causes of cancer-related deaths worldwide, patients with lung cancer often require invasive monitoring or treatment and have a relatively low survival rate [17], especially those in ICU [4]. In the present study, the in-hospital mortality rate of lung cancer patients in the ICU was 19.92%. Peng et al. demonstrated that the in-hospital mortality rate for ICU patients with lung cancer was 26.0% in the original cohort and 26.4% in the validation cohort [18]. Our findings indicated that high WHR was associated with increased in-hospital mortality in patients with lung cancer and length of stay in ICU. Additionally, a high WHR was also related to a higher risk of in-hospital mortality in patients with higher APACHE score, in male patients, and in patients receiving ventilation.

We observed that a high WHR level was associated with a high risk of in-hospital mortality in ICU patients with lung cancer. A study investigating the value of new preoperative WHR for patients with gastric adenocarcinoma found that patients with an increased WHR had a significantly decreased 5-year OS [8]. A study by Shen et al. reported that preoperative WHR is an effective prognostic indicator for hepatocellular carcinoma in patients undergoing curative hepatectomy [14]. A high WHR represents a high WBC count and a low level of HGB. Generally, an elevated WBC indicates a compromised immune system [9]. Previous studies have suggested that a high WBC count is associated with increased total and cardiovascular mortality [19, 20]. The association between WBC and mortality in cancer has also been reported. The result from a previous study revealed that an increased WBC level was positively associated with all-cause mortality, specifically correlating with cancer, in all populations, including the elderly [21]. There was evidence that the association between WBC counts and prostate cancer mortality was stronger with a longer follow-up time [22]. A decrease in HGB can lead to tumor hypoxia, which stimulates tumor growth by stimulating angiogenesis, acquiring genome mutations, and increasing resistance to apoptosis, and further leads to increased staging and a poor prognosis [12]. On the other hand, tumor-related inflammation may lead to the release of various inflammatory factors, which may interfere with erythropoietin synthesis and lead to a decrease in HGB [23]. WHR is a readily available parameter and can be calculated clinically by the WBC to HGB ratio. Our results highlight the importance of blood cell count parameters in monitoring the outcome of ICU patients with lung cancer. The prognosis of ICU patients with lung cancer may require prompt attention when the WHR is elevated or the HGB is decreasing. Early attention to at-risk populations may also contribute to timely intervention in the future.

In this study, a high WHR was associated with a higher risk of in-hospital mortality in patients with a higher APACHE score. APACHE-II has been widely used in clinical practice due to its dependability and convenience and the higher the score is, the higher the mortality and poorer prognosis of the patient [24]. Shen et al. found that an APACHE II score < 16 resulted in the lowest 28-day and 90-day mortality in predefined do-not-intubate lung cancer patients [25]. In addition, a high WHR was related to a higher risk of in-hospital mortality in ICU lung cancer patients receiving mechanical ventilation. Shin et al. reported that the 28-day mortality in advanced lung cancer patients receiving mechanical ventilation at the emergent department was poor [26]. A study by Soubani et al. demonstrated that among lung cancer patients admitted to the ICU, the need for mechanical ventilation was a clinical factor in predicting hospital mortality [27]. A higher APACHE score or receiving mechanical ventilation in ICU lung cancer patients may represent a more severe type of cancer. The in-hospital mortality in lung cancer patients admitted to ICU is a discrepancy according to the lung cancer stage [4]. Our finding may imply the vital of WHR in more severe ICU patients with lung cancer. Furthermore, an increased WHR was associated with a higher risk of in-hospital mortality in male ICU patients with lung cancer. Previous studies have confirmed that the mortality rate of male lung cancer patients is higher than females [28, 29]. Gender differences in the histological types and developmental stages on diagnosis may partially explain the bad prognosis of male lung cancer patients [30]. The association between WHR and in-hospital mortality of lung cancer in ICU may vary by populations.

To the best of our knowledge, this is the first study to investigate the prognostic utility of WHR for cancer patients in the ICU. These ICU lung cancer patients tend to have poor long-term survival and higher economic costs. Investigating the association between biomarkers and the prognosis of patients with lung cancer in the ICU may be important for the management of lung cancer in the ICU. In addition, WHR is an easily available parameter, thus this study provides a useful reference for clinicians to confirm biomarkers associated with prognostic for ICU patients with lung cancer. However, this study has several limitations. Firstly, this study was a retrospective cohort study and is therefore subject to the typical bias associated with this type of data collection. Secondly, due to the limitations of the database, there was a lack of tumor stage and other parameters that may affect prognosis. However, this study reflected the body condition and severity of lung cancer patients by considering APACHE score and other parameters, and evaluated the applicability of WHR in different populations. Thirdly, this study focused on the association between WHR at baseline (admission to ICU) and in-hospital mortality of ICU patients with lung cancer, without considering the possible changes in WHR and their effects during hospitalization. Further prospective studies should be conducted to explore the association between dynamic changes in WHR and prognosis in ICU patients with lung cancer.

## Conclusions

This study suggests a higher level of WHR was related to the risk of in-hospital mortality in ICU patients with lung cancer, especially in males and in those with a higher APACHE score and who received mechanical ventilation. More attention should be paid to the population with high WHR levels, which may be beneficial to the prognosis of ICU patients with lung cancer.

### Supplementary Information

**Additional file 1: Supplementary Table 1.** Sensitivity analysis before and after interpolation

## Data Availability

The datasets generated and/or analyzed during the current study are available in the eICU Database, https://eicu-crd.mit.edu/.

## Abbreviations

ICU: Intensive care unit
PLR: Platelet to lymphocyte ratio
NLR: Neutrophil to lymphocyte ratio
WBCs: White blood cells
HGB: Hemoglobin
WHR: WBC to HGB ratio
Eicu: Electronic ICU
BMI: Body mass index
SBP: Systolic blood pressure
DBP: Diastolic blood pressure
CAD: Coronary artery disease
CHF: Congestive heart failure
AF: Atrial fibrillation
RF: Renal failure
APACHE: Acute Physiology and Chronic Health Evaluation
BUN: Blood urea nitrogen
RRT: Rapid resolution therapy
ICD-9/10: Ninth or tenth revision of the International Classification of Diseases
