# Supplementary material for: Association between white blood cell count to hemoglobin ratio and risk of in-hospital mortality in patients with lung cancer
Source: BMC Pulm Med. 2023 Aug 18;23:305. doi: 10.1186/s12890-023-02600-7 (PMC10436509; doi:10.1186/s12890-023-02600-7)
Supplement: Supplementary file 1 — Additional file 1: Supplementary Table 1. Sensitivity analysis before and after interpolation [file 12890_2023_2600_MOESM1_ESM.docx]

Supplementary Table 1 Sensitivity analysis before and after interpolation

| Variables | Random forest interpolation | | *P* |
| --- | --- | --- | --- |
|  | Before (N=768) | After (N=768) |  |
| Race, n (%) |  |  | 1.000 |
| White | 633 (84.1%) | 639 (84.2%) |  |
| Other | 120 (15.9%) | 120 (15.8%) |  |
| Heart rate, times/minute, M (Q_1_, Q_3_) | 98.0 (82.0, 115) | 99.0 (82.0, 115) | 0.838 |
| BMI, kg/m^2^, M (Q_1_, Q_3_) | 25.1 (21.8, 29.6) | 25.3 (21.8, 29.8) | 0.750 |
| Respiratory rate, breaths/minute, M (Q_1_, Q_3_) | 20.0 (17.0, 25.0) | 20.0 (17.0, 25.0) | 0.982 |
| SBP, mmHg, M (Q_1_, Q_3_) | 120 (100, 140) | 119 (100, 140) | 0.836 |
| DBP, mmHg, M (Q_1_, Q_3_) | 68.0 (56.0, 80.0) | 68.0 (56.5, 80.0) | 0.991 |
| Temperature, °C, M (Q_1_, Q_3_) | 37.0 (36.0, 37.0) | 37.0 (36.0, 37.0) | 0.913 |
| RDW, %, M (Q_1_, Q_3_) | 15.6 (14.0, 17.7) | 15.6 (14.0, 17.7) | 1.000 |
| Creatinine, mg/dL, M (Q_1_, Q_3_) | 0.90 (0.69,1.30) | 0.90 (0.69, 1.30) | 0.989 |
| BUN, mg/dL, M (Q_1_, Q_3_) | 19.0 (13.0, 29.0) | 19.0 (13.0, 29.0) | 0.943 |
| Glucose, mmol/L, M (Q_1_, Q_3_) | 133 (106, 168) | 133 (106, 168) | 0.936 |
| Bicarbonate, mmol/L, M (Q_1_, Q_3_) | 25.0 (22.0, 28.0) | 25.0 (22.0, 28.0) | 0.948 |
| Sodium, mmol/L, M (Q_1_, Q_3_) | 137 (134, 140) | 137 (134, 140) | 0.990 |
| Potassium, mmol/L, M (Q_1_, Q_3_) | 4.10 (3.70, 4.50) | 4.10 (3.70, 4.50) | 0.987 |
| Chloride, mmol/L, M (Q_1_, Q_3_) | 101 (97.0, 105) | 101 (97.0, 105) | 0.990 |
| Apache score, M (Q_1_, Q_3_) | 58.0 (46.0, 75.0) | 58.0 (46.0, 75.0) | 0.906 |

Notes: BMI: body mass index; SBP: systolic blood pressure; DBP: diastolic blood pressure; RDW: red cell distribution width; BUN: blood urea nitrogen; Mean ± SD: means +- standard deviation; M: Median; Q_1_: 1st Quartile; Q_3_: 3st Quartile.
